# A chromosome-level genome of the striated frogfish (*Antennarius striatus*)

**DOI:** 10.1038/s41597-024-03514-7

**Published:** 2024-06-21

**Authors:** Mingliang Hu, Deqian Fan, Jiaqi Hao, Fenghua Zhang, Wenjie Xu, Chenglong Zhu, Kun Wang, Xiaojing Song, Lisen Li

**Affiliations:** 1 Shaanxi Key Laboratory of Qinling Ecological Intelligent Monitoring and Protection, School of Ecology and Environment, Northwestern Polytechnical University, Xi’an, 710129 China; 2 East China Sea Fisheries Research Institute, Chinese Academy of Fishery Sciences, Shanghai, 200090 China

**Keywords:** Genome, Evolution

## Abstract

The striated frogfish (*Antennarius striatus*), a member of the sub-order Antennarioidei within the order Lophiiformes, possesses remarkable adaptations. These include the ability to modulate body coloration for camouflage, utilize bioluminescent esca for predation, and employ elbow-like pectoral fins for terrestrial locomotion, making it a valuable model for studying bioluminescence, adaptive camouflage, fin-to-limb transition, and walking-like behaviors. To better study and contribute to the conservation of the striated frogfish, we obtained the micro-CT image of the pectoral fin bones and generated a high-quality, chromosome-level genome assembly using multiple sequencing technologies. The assembly spans 548.56 Mb with a contig N50 of 21.05 Mb, and 99.35% of the genome is anchored on 24 chromosomes, making it the most complete genome available within Lophiiformes. The genome annotation revealed 28.43% repetitive sequences and 23,945 protein-coding genes. This chromosome-level genome provides valuable genetic resources for frogfish conservation and offers insights into the genetic mechanisms underlying its unique phenotypic evolution. Furthermore, it establishes a foundation for future research on limb development and adaptive camouflage in this species.

## Background & Summary

The striated frogfish is a member of Antennarioidei, the representative of Lophiiformes on coral reefs^1^. Known for its unique appearance, the defining feature of the striated frogfish lies in the morphology of its first adapted dorsal-fin spine (esca), which is often bioluminescent^1^ and positioned at the tip of the snout. This adaptation functions as a lure to attract prey through aggressive mimicry^1–3^ and has driven the order’s diversification^4^. Moreover, species of the genus Antennarius possess the fastest feeding speed among animals, expanding their buccal cavity and devouring prey in less than 4 ms^5^. To better adapt to its environment, the striated frogfish has the ability to change its color and pigmentation within a few weeks^6^, a trait shared with other frogfishes^7–9^. Another remarkable adaptation of the striated frogfish is its walking-like behavior, which is exhibited by certain fish using the bipedal function of their fins^10–16^. This behavior represents an independent fin-to-limb transition^17,18^, as walking is the primary mode of locomotion for terrestrial vertebrates^19^ and evolved with the development of limbs^10^. The modified pectoral fins of Antennarius resemble an elbow-like appearance, allowing these fish to walk along the substrate^2,16,18^, further highlighting the unique adaptations of this species.

In addition to shared characteristics, the striated frogfish possesses some unique features. Unlike the near-smooth surfaces of the other members in the Antennarioidei order, the striated frogfish is covered with dermal spinules that resemble hairs, enabling better camouflage and slowing down the movement of the prey^6,20^. The body of the striated frogfish is usually more elongated than other frogfish species and is typically covered in distinctive dark stripes or bands that give it its name^7,8^, allowing it to hide more effectively on the sea floor. The survival of the striated frogfish is also greatly challenged by the decline of coral reefs due to climate change and rising sea temperatures.

Although many physiological and behavioral studies have been conducted on the adaptive camouflage and walking-like behaviors of frogfishes^1,3,7,10^, genetic studies on this uniquely characterized taxon of frogfish remain scarce due to the lack of high-quality genomic data. In this study, we provided the Micro-CT image of the pectoral fin bones, and a chromosome-level draft genome (~548.56 Mb) of the striated frogfish with a contig N50 of 21.05 Mb by employing advanced PacBio SMRT Circular Consensus Sequencing (CCS), high-throughput chromosome conformation capture (Hi-C) sequencing, and next-generation sequencing (NGS). The sequencing read mapping rate, completeness of BUSCO conserved genes, gap number, anchoring rate of chromosomes, contig N50, and genome synteny collectively demonstrate the continuity and accuracy of this genome assembly, making it the highest quality Lophiiformes genome assembly to date. In conclusion, this high-quality genome serves as a valuable resource for future studies of fish evolution, particularly in the areas of bioluminescence, adaptive camouflage, fin-to-limb transition, and walking-like behaviors.

## Methods

### Sample collection, morphological detection, and library construction

**Fig. 1:**
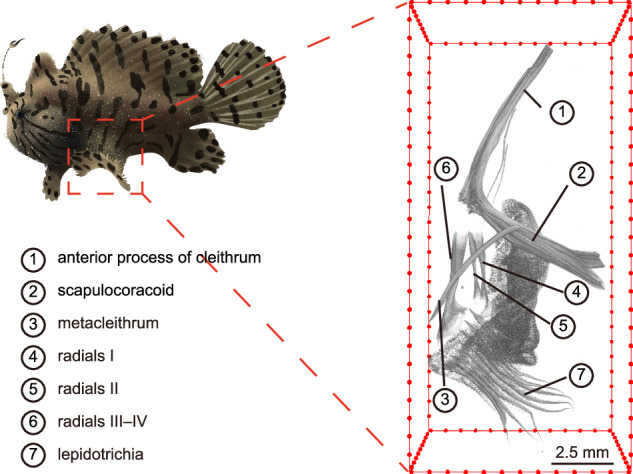
Morphology of the striated frogfish. Photograph and Micro-CT image of pectoral fin bones of the striated frogfish.

Adult striated frogfish were collected and photographed from the East China Sea at the coordinate of 31°29′ N, 125°33′ E during the fishery resources investigations (2022.9.9). After anesthetizing this fish, we collected the tissue samples including muscle, kidney, gill, heart, eyes, swimbladder, liver, skin, stomach, tongue, pelvic fin, pectoral fin, and caudal fin. These samples were frozen in liquid nitrogen and then stored at −80 °C. Additionally, the pectoral fin was fixed in a 4% paraformaldehyde solution for 48 hours and then washed three times with phosphate-buffered saline (PBS). Subsequently, it was stored in a 75% ethanol solution at 4 °C. The processed pectoral fin was scanned with a SkyScan 1176 small animal micro-CT scanner (BRUKER Corporation, Germany) at 18 µm, and NRecon was used to reconstruct the three-dimensional image. The type of pectoral fin bone, including the anterior, process of cleithrum, scapulocoracoid, metacleithrum, radials I-IV, and lepidotrichia, was defined based on previous studies^18^ (Fig. 1).

For genomic DNA extraction, the liver tissue sample was used for PacBio SMRT sequencing, while the kidney tissue samples were used for both Next-Generation sequencing and Hi-C sequencing. Additionally, various tissues including muscle, kidney, gill, heart, eyes, swimbladder, liver, skin, stomach, tongue, pelvic fin, pectoral fin, and caudal fin were used for bulk-RNA sequencing. All experimental procedures and animal use for this study complied with all relevant ethical regulations and were approved by the Northwestern Polytechnic University Ethics Committee Institutional Review Board (202101025).

The Illumina sequencing library was prepared using the NEB Next® Ultra™ DNA Library Prep Kit (NEB, USA), sequenced on the Illumina NovaSeq. 6000 platform, generating 150 bp paired-end reads with an insert size of 350 bp. Raw sequencing reads were filtered for adapter sequences, low-quality reads, and trimmed using fastp v0.20^21^ with default parameters and yielding a total of 139.22 Gb of clean short reads. To generate HiFi reads, we followed the standard protocol of PacBio (Pacific Biosciences, CA, USA), which yielded 36.47 GB of clean long reads using the PacBio Sequel II platform.

For Hi-C library construction, we followed the standard protocol described in a published study^22^. The fresh kidney tissue was fixed with 2% formaldehyde to maintain both intra- and intermolecular interactions. The cross-linked DNA was then digested with the restriction enzyme MboI. The resulting sticky ends were biotinylated by incubation with biotin-14-dATP and the Klenow fragment of DNA polymerase I. Following DNA purification and the removal of biotin from unligated ends, the Hi-C products were enriched and physically sheared to fragment sizes of 200–300 base pairs. The biotin-tagged Hi-C DNA was subsequently isolated and processed into paired-end sequencing libraries. Sequencing was performed on the HiSeq X Ten platform, yielding a total of 110.64 Gb of cleaned reads using fastp v0.20^21^.

Following the instructions, total RNA was extracted using the TRlzol reagent (Life Technologies, California, USA). After library construction for each sample, 150 bp paired-end reads were generated using the Illumina NovaSeq 6000 platform. Raw data was cleaned using fastp v0.20^21^ and more than 6 Gb clean paired-end reads was generated for each tissue (supplementary Table. S1).

### Chromosome-level genome assembly of the striated frogfish

**Fig. 2:**
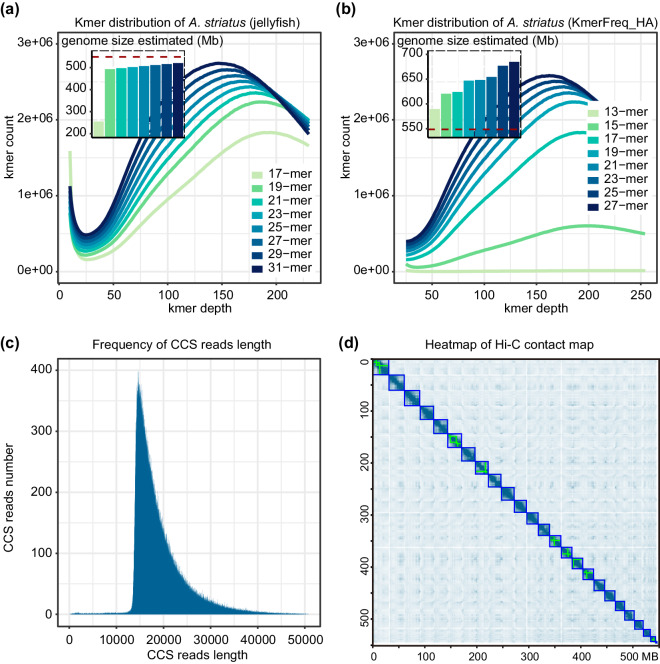
Genome assembly of the striated frogfish. (**a**) Different *k*-mer distribution (*k* = 17, 19, 21, 23, 25, 27, 29, 31) of the striated frogfish genome using jellyfish. The range of genome size is estimated from 255.02 Mb to 520.49 Mb. The red dashed line indicates the size of the assembled genome of striated frogfish (548.56 Mb). (**b**) Different *k*-mer distribution (*k* = 13, 15, 17, 19, 21, 23, 25, 27) of the striated frogfish genome using KmerFreq_HA. Genome size inferred by k num divided by k depth. The estimated range of genome size for the striated frogfish is 589.74 Mb to 684.90 Mb. The red dashed line indicates the size of the assembled striated frogfish genome (548.56 Mb). (**c**) Length distribution of circular consensus sequence (CCS) reads for the striated frogfish. (**d**) Hi-C linkage density heat map of the striated frogfish. The x- and y-axes represent genomic positions.

To estimate the genome size, we performed *k*-mer analysis based on Illumina sequencing reads using two methods, Jellyfish v2.2.10^23^ and KmerFreq_HA v2.0^24^, to ensure the accuracy and reliability of the results, respectively. Jellyfish v2.2.10^23^ was used to calculate the *k*-mer distribution frequency, and GenomeScope v2.0^25^ calculated the optimal *k*-mer values and estimated the corresponding genome sizes. We used various *k*-mer values (17, 19, 21, 23, 25, 27, 29, 31), and the corresponding genome sizes were 255.02 Mb, 492.93 Mb, 497.45 Mb, 502.28 Mb, 506.82 Mb, 511.24 Mb, 515.74 Mb, and 520.49 Mb respectively (Fig. 2a) Additionally, KmerFreq_HA v2.0^24^ calculated the kmer distribution frequency, and manually selected the optimal *k*-mer values and estimated the corresponding genome sizes. Various *k*-mer values (13, 15, 17, 19, 21, 23, 25, 27) were used, and the corresponding genome sizes were 589.74 Mb, 620.53 Mb, 624.24 Mb, 647.03 Mb, 648.44 Mb, 654.50 Mb, 677.02 Mb, and 684.90 Mb, respectively (Fig. 2b).

Based on the PacBio SMRT sequencing technology, a total of 1,957,851 HiFi reads were obtained. More than 99.69% of the reads were longer than 13 Kb, and the N50 value was greater than 18 Kb. The distribution of these reads is shown in Fig. 2c. The raw genome assembly, with a size of 555.05 Mb, was assembled by hifiasm v0.16.1^26^ with default parameters. Purge_dups v1.2.5^27^ was used for genome de-redundancy based on HiFi reads and Illumina reads, and the final contig-level genome spans 548.56 Mb, with 54 contigs and an N50 of 21.05 Mb (Table 1). The reads of Hi-C seq were mapped to the contig-level genome using BWA v0.7.17-r1198-dirty^28^ with default parameters. Then yahs v1.1a-r3^29^ were used to generate the scaffold genome assembly. Based on the strength of chromatin interactions, JuiceBox v.1.11.08^30^ was used for visual correction of the assembly, and the contigs with no significant interaction with other contigs were considered as separate scaffolds. The contigs allocated to a chromosome are connected with 200 ‘N’ to construct the final chromosome-level genome assembly. The final chromosome-level assembly, with the size of 548.56 Mb and an contig N50 of 21.05 Mb (Table 1), contained 24 chromosomes with an anchoring rate of 99.35%, and the number of chromosomes was consistent with the warty frogfish^31^ (*Antennarius maculatus*, GCA_013358685.1, NCBI), the member in the same genus, Antennarius (Fig. 2d).

**Table 1:** Statistics of the genome assembly.

Term	Contig assembly		Hi-C assembly	
	Size (bp)	Number	Size (bp)	Number
N90	7,734,013	26	18,307,533	21
N80	13,085,921	21	21,047,747	18
N70	16,841,418	17	21,220,182	16
N60	18,982,356	14	21,998,917	13
N50	21,047,747	11	23,897,462	11
Max length (bp)	30,347,754	—	31,208,511	—
Total size (bp)	548,559,420	—	548,562,020	—
Total number	—	48	—	42

### Assessment of the genome assembly

The reads sequenced by different sequencing methods were mapped to the genome. SAMtools v1.16.1^32^ was used to convert the alignment file format from sam to bam. Minimap2 v2.17^33^ was used to map the HiFi reads to the genome. The Next-Generation DNA sequencing reads were mapped to the genome by bwa-mem2 v2.2.1^34^. The HiFi reads and next-generation DNA reads reply rates were 99.99% and 100.00%, respectively, covering 99.97% and 99.99% of the genome, (Table. S1). Using hisat2 v2.2.0^35^ to align the RNA sequencing reads to the genome, the alignment rate ranged from 95.99% to 98.83%, except for the 78.85% alignment rate of skin (Table. S1).

**Fig. 3:**
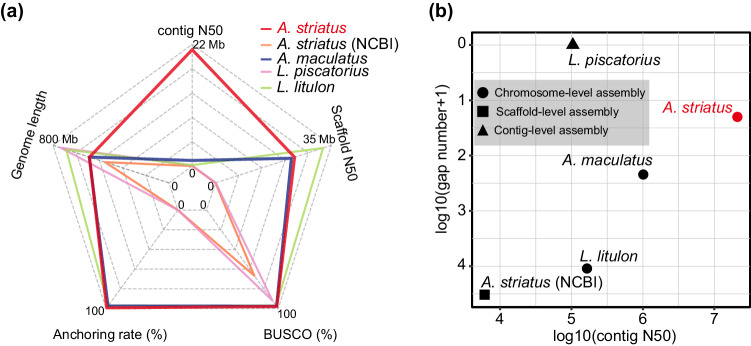
Quality statistics of Lophiiformes genomes. (**a**) The five dimensions represent the contig N50, Scaffold N50, the BUSCO scores based on actinopterygii_odb10 (2024.1.8), the anchoring rate of chromosomes, and the length of the genome assembly, respectively. (**b**) The x-axes indicate the contig N50 of the genome assembly, while the y-axes indicate the gap number of the genome assembly.

Genome completeness was evaluated using BUSCO v5.4.3^36^ and the actinopterygii_odb10 database (version 2024.01.08), and the assembled genome has 98.0% completeness of BUSCO conserved genes, including 96.7% single-copy orthologous genes. The gap number, anchoring rate, contig N50 length, scaffold N50 length, and whole genome length of the genome were counted by proprietary Perl scripts (https://github.com/bright-hu/Antennarius_striatus/tree/main/05.code/02.genome_assessment,). We compared the genome quality by the contig N50 length, BUSCO score, gap number, and anchoring rate with 4 published Lophiiformes genomes, including the striated frogfish^37^ (GCA_900303275.1, NCBI), the warty frogfish (GCA_013358685.1, NCBI), *Lophius piscatorius*^38^ (GCA_009660295.1, NCBI), and *Lophius litulon*^39^ (CNA0007339, CNGBdb). The scaffold N50 of the striated frogfish (23.9 Mb) and the warty frogfish (22.9 Mb) were proximate. Except for the gap number of *Lophius piscatorius* genome at the contig level, the genome assembly is the best in all statistical parameters (Fig. 3).

**Fig. 4:**
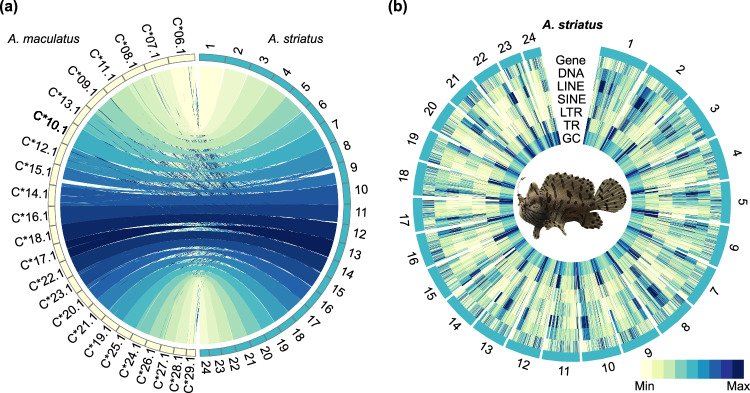
Overview of the striated frogfish genome. (**a**) Synteny alignment in striated frogfish (*Antennarius striatus*) and warty frogfish (*Antennarius maculatus*). (**b**) Circos plot indicating gene density, repetitive sequences (including DNA elements, LINE, SINE, LTRs, and TRs) density, and GC content of the striated frogfish genome assembly.

Moreover, to evaluate the assembly continuity, we performed a genome alignment between the striated frogfish and the same genus member, the warty frogfish, using LAST v1080^40^ to identify syntenic regions. The alignment results showed intact synteny and pairwise chromosome correspondence between the two genomes (Fig. 4a), further confirming the quality and accuracy of the striated frogfish genome assembly.

### Genome annotation

Comprehensive annotation of the striated frogfish genome was performed, including repeat sequences, protein-coding genes, and functional prediction. For the annotation of repetitive sequences, Tandem Repeats Finder (TRF) v4.09^41^ and RepeatModeler v1.0.11 (https://www.repeatmasker.org/RepeatModeler/) were used for *de novo* annotation, and RepeatMasker v4.0.7^42^ and RepeatProteinMask v1.0.8^42^ were applied for performing homolog-based annotation. Then the repetitive sequences annotated were soft-masked in the genome using the maskFastaFromBed function of BEDTools v2.28.0^43^. Finally, approximately 155.96 Mb of repetitive regions were annotated, accounting for 28.43% of the whole genome, including 35.20 Mb of tandem repeats (TRs), 15.73 Mb of long terminal repeats (LTRs), 7.84 Mb of short interspersed nuclear elements (SINEs), 42.61 Mb of long interspersed nuclear elements (LINEs), and 48.55 Mb of DNA elements (Fig. 4b).

Combined methods were used for predicting protein-coding genes, including *de novo* prediction, species’ protein-based homology prediction, and transcripts-based prediction. Augustus v2.5.5^44^ was used for *de novo* prediction. For species’ protein-based homology prediction, we collected protein sequences from several species, including zebrafish (GRCz11, Ensembl), Japanese medaka (ASM223471v1, Ensembl), Three-spined stickleback (BROADS1, Ensembl), Large spiny eel (fMasArm1.2, Ensembl), human (GRCh38, Ensembl), mouse (GRCm38, Ensembl), and *Lophius litulon* (CNA0007339, CNGBdb). Miniprot v0.12^45^ was used for predicting gene structures based on the homology proteins. For transcript-based prediction, SPAdes v3.14.0^46^ and TransDecoder v5.5.0 (https://github.com/TransDecoder/) were used to assemble the transcripts and predict protein structure for each sample based on RNA sequencing data. These proteins were aligned to the genome of the striated frogfish by BLAT v35.1^47^, and GeneWise v2.4.1^48^ was employed to predict the gene structures. Finally, EVidenceModeler v1.1.1^49^ was used to integrate the above results into a gene set, and a total of 23,945 protein-coding genes were annotated, with 98.1% complete BUSCO conserved genes of actinopterygii_odb10 (version 2024.01.08) (Table 2).

**Table 2:** Statistics of the genome assembly and annotated proteins for the presence of conserved BUSCO orthologs.

actinopterygii_odb10 (2024.1.8)	Genome	Protein
Complete BUSCOs (C)	3,568	3,573
Complete and single-copy BUSCOs (S)	3,519	3,506
Complete and duplicated BUSCOs (D)	49	67
Fragmented BUSCOs (F)	10	14
Missing BUSCOs (M)	62	53
Total BUSCO groups searched	3,640	3,640
Summarize	98.0%	98.1%

Functional annotation of the protein-coding gene set was performed using InterPro v5.44–79.0^50^ and the public databases, including Gene Ontology (GO) annotations (http://geneontology.org/), Kyoto Encyclopedia of Genes and Genomes (KEGG: https://www.kegg.jp/), Swiss-Prot (https://www.uniprot.org), TrEMBL (https://www.uniprot.org), Cluster of Protein Orthologous Groups (COG: https://www.ncbi.nlm.nih.gov/COG/), and non-redundant proteins (NR: https://ftp.ncbi.nlm.nih.gov/blast/db). 21,543 genes, representing 89.97% of all genes, were annotated with at least one term (Table 3).

**Table 3:** Statistics of the functional annotation of protein-coding genes.

Term	Number	Percentage (%)
InterPro	19,674	82.16
GO	13,682	57.14
KEGG	16,635	69.47
Swissprot	20,188	84.31
TrEMBL	21,195	88.52
COG	7,989	33.36
NR	21,377	89.28
Annotated genes	21,543	89.97
Non-annotated genes	2,402	10.03
Total genes	23,945	—

## Data Records

The DNA and RNA sequencing data have been deposited in the NCBI Sequence Read Archive (SRA) database, including SRR28026916^51^, SRR28026917^52^, SRR28026918^53^, SRR28038112^54^, SRR28038113^55^, SRR28038114^56^, SRR28038115^57^, SRR28038116^58^, SRR28038117^59^, SRR28038118^60^, SRR28038119^61^, SRR28038120^62^, SRR28038121^63^, SRR28038122^53^, SRR28038123^64^, SRR28038124^65^, which is associated with the BioProject accession number PRJNA1078610. The draft genome assembly of the striated frogfish has been submitted to the NCBI GeneBank with the accession number JBANDW000000000^66^. The draft genome assembly and genome annotation were deposited in the *Figshare* database (10.6084/m9.figshare.25340587)^67^.

## Technical Validation

The size of the genome assembly, 548.56 Mb, was between the genome size estimated by jellyfish v2.2.10 (255.02~520.49 Mb) and KmerFreq_HA v2.0 (589.74 ~ 684.90 Mb). The quality of the genome assembly was assessed in four steps. Initially, we calculated the mapping rate and genome coverage rate to measure the accuracy of the genome assembly. The mapping rates for HiFi reads and Next-Generation DNA reads were 99.99% and 100.00%, respectively, and covered 99.97% and 99.99% of the genome. The alignment rate of the RNA sequencing reads ranged from 95.99% to 98.83%, except for the 78.85% alignment rate of the skin sample. Moreover, completeness was assessed using BUSCO v5.4.3 based on the actinopterygii_odb10 (version 2024.01.08) database (n = 3,640). The completeness of BUSCOs in the final genome assembly was 98.0%, including 3,519 (96.6%) single-copy BUSCOs and 49 (1.3%) duplicate BUSCOs. In addition, we further evaluated the genomic metrics of the published Lophiiformes genomes. The final genome assembly was the best genome in the Lophiiformes, with the longest contig N50 (21.05 Mb), the best completeness of BUSCOs (98.0%), the highest chromosome anchoring rate (99.35%), and the fewest gaps (19). Ultimately, we performed a genome alignment between the striated frogfish and the same genus member, the warty frogfish. Intact synteny and pairwise chromosome correspondence further confirmed the quality and accuracy of the striated frogfish genome assembly.

### Supplementary information

Table. S1. Statistics of sequencing data and matching rate to genome

## Data Availability

Softwares for data analyses were mentioned in Methods. The core code and parameters are available at https://github.com/bright-hu/Antennarius_striatus/.
